# Impact of efalizumab on patient-reported outcomes in high-need psoriasis patients: results of the international, randomized, placebo-controlled Phase III Clinical Experience Acquired with Raptiva (CLEAR) trial [NCT00256139]

**DOI:** 10.1186/1471-5945-5-13

**Published:** 2005-12-16

**Authors:** Jean-Paul Ortonne, Neil Shear, Stephen Shumack, Eric Henninger, the CLEAR Multinational Study Group

**Affiliations:** 1 Hôpital L'Archet, Nice, France; 2 Ventana Clinical Research, Toronto, Ontario, Canada; 3 St George Dermatology, Kogarah, Australia; 4 Serono International S.A., Geneva, Switzerland; 5 A complete list of the principal investigators can be found at the end of this article

## Abstract

**Background:**

Chronic psoriasis can negatively affect patients' lives. Assessing the impact of treatment on different aspects of a patient's health-related quality of life (HRQOL) is therefore important and relevant in trials of anti-psoriasis agents. The recombinant humanized IgG_1 _monoclonal antibody efalizumab targets multiple T-cell-dependent steps in the immunopathogenesis of psoriasis. Efalizumab has demonstrated safety and efficacy in several clinical trials, and improves patients' quality of life. Objective: To evaluate the impact of efalizumab on HRQOL and other patient-reported outcomes in patients with moderate to severe plaque psoriasis, including a large cohort of High-Need patients for whom at least 2 other systemic therapies were unsuitable because of lack of efficacy, intolerance, or contraindication.

**Methods:**

A total of 793 patients were randomized in a 2:1 ratio to receive efalizumab 1 mg/kg/wk (n = 529) or placebo (n = 264) for 12 weeks. The study population included 526 High-Need patients (342 efalizumab, 184 placebo). The treatment was evaluated by patients using the HRQOL assessment tools Short Form-36 (SF-36) and Dermatology Life Quality Index (DLQI). Other patient-reported assessments included the Psoriasis Symptom Assessment (PSA), a visual analog scale (VAS) for itching, and the Patient's Global Psoriasis Assessment (PGPA).

**Results:**

Efalizumab was associated with improvements at Week 12 from baseline in patient-reported outcomes, both in the total study population and in the High-Need cohort. Among all efalizumab-treated patients, the DLQI improved by 5.7 points from baseline to Week 12, relative to an improvement of 2.3 points for placebo patients (*P *< .001). Corresponding improvements in DLQI in the High-Need cohort were 5.4 points for efalizumab compared to 2.3 for placebo (*P *< .001). Improvements from baseline on the SF-36, PSA, PGPA, and itching VAS at Week 12 were also significantly greater in efalizumab-treated patients than for placebo.

**Conclusion:**

A 12-week course of efalizumab improved HRQOL and other patient-reported outcomes in patients with moderate to severe plaque psoriasis. The benefits of efalizumab therapy in High-Need patients were similar to those observed in the total study population, indicating that the beneficial impact of efalizumab on QOL is consistent regardless of disease severity, prior therapy, or contraindications to previous therapies.

## Background

Psoriasis is an incurable, chronic, immune-mediated disease. The impact of psoriasis on patients' physical, social, and psychological functioning and health-related quality of life (HRQOL) has been well documented [1-4]. Many patients report moderate to extreme feelings of anxiety, anger, and depression [5]. Increasing severity of psoriasis appears to correlate closely with increased severity of depression and, in turn, with higher frequency of suicidal ideation [6,7]. However, disease severity as measured by instruments such as the Psoriasis Area and Severity Index (PASI) is not the sole factor determining the burden of illness, because relatively minor psoriasis located on visible parts of the body may also have a detrimental effect on HRQOL [8].

Quality-of-life indicators and traditional physician-assessed clinical outcomes are correlated only weakly in psoriasis, suggesting that they measure separate, complementary aspects of the disease's impact [9,10]. Thus, assessment of HRQOL during clinical trials is important for establishing the overall impact of an investigational anti-psoriasis agent as well as its likelihood of acceptance by the psoriatic population [11].

The negative impact of psoriasis on patients' HRQOL is compounded by side effects and toxicities associated with many current systemic psoriasis treatments – which may also increase the need for laboratory monitoring – and inconvenient administration regimens. Such issues highlight the need for new therapies that provide greater safety, efficacy, and convenience than those currently available. An improved understanding of the critical role of the immune system, in particular that of T cells, in the pathogenesis of psoriasis has provided the impetus for the development of biological agents that target particular steps in the immunopathogenesis of the disease [12].

Efalizumab is a humanized monoclonal antibody that blocks multiple T-cell-dependent functions implicated in the pathogenesis of psoriasis [13]. Multiple placebo-controlled Phase III clinical studies have assessed the safety and efficacy of efalizumab therapy in patients with moderate to severe chronic plaque psoriasis. These studies demonstrated a favorable safety profile as well as efficacy versus placebo according to physician-assessed measures, such as improvement from baseline in PASI [14-19]. Furthermore, in these trials, efalizumab was associated with significantly greater improvements from baseline versus placebo in all patient-reported outcome measures, including improvements in HRQOL as measured by instruments such as the Dermatology Life Quality Index (DLQI) [20].

The international Phase III trial (the Clinical Experience Acquired with Raptiva^® ^[CLEAR] study) evaluated the safety and efficacy of efalizumab in patients with moderate to severe plaque psoriasis, including a large cohort of High-Need patients, defined as those for whom at least 2 other systemic therapies were unsuitable because of lack of efficacy, intolerance, or contraindication. As reported elsewhere, efalizumab treatment was associated with significant improvements versus placebo in physician-assessed outcome measures and demonstrated a favorable safety profile over 12 weeks of therapy (Dubertret L, Sterry W, Bos JD, *et al.*, unpublished data.) The effects of efalizumab on HRQOL and other patient-reported outcomes in the first 12-week treatment period of this trial are described here.

## Methods

### Study design

This Phase III multiphase, randomized, double-blind, placebo-controlled, parallel-group multicenter trial was conducted to evaluate the safety and efficacy of weekly efalizumab compared with placebo in patients with moderate to severe plaque psoriasis. During the study period, but before results were available, a protocol amendment restricted enrollment to patients meeting the definition of "High-Need" patients (Dubertret L, Sterry W, Bos JD, *et al.*, unpublished data). High-Need patients were subjectively defined as those for whom 2 or more current systemic therapies (e.g., photochemotherapy [PUVA], cyclosporine, corticosteroids, methotrexate, oral retinoids, mycophenolate mofetil, thioguanine, hydroxyurea, sirolimus, azathioprine, 6-mercaptopurine) were ineffective, poorly tolerated, or contraindicated.

During the initial 12-week treatment period, eligible patients were randomized in a 2:1 ratio to receive either efalizumab 1 mg/kg (Raptiva^®^, Genentech, Inc.) or matching placebo, administered subcutaneously once weekly for 12 weeks (Dubertret L, Sterry W, Bos JD, *et al.*, unpublished data). Patients were initially randomized between March 12, 2003, and June 3, 2003; following the protocol amendment, patients were randomized between July 16, 2003, and September 30, 2003. A conditioning dose of 0.7 mg/kg was administered on Day 0; subsequent doses were administered at 1 mg/kg. Systemic therapies for psoriasis were discontinued at least 28 days before treatment initiation, and topical therapies were discontinued 14 days before treatment initiation. Other systemic psoriasis therapies or phototherapy were not permitted during the trial. Emollients and tar or salicylic acid preparations for scalp lesions and small quantities of group VI or VII topical corticosteroids for lesions on the face, hands, feet, groin, or axillae were permitted, but these medications were not to be used on the day of a scheduled PASI assessment.

A total of 104 clinical centers in Europe, Russia, Israel, Australia, Mexico, and Canada participated in this trial. The relevant ethics committee at each participating study center reviewed and approved the study protocol, and all patients provided written informed consent before undergoing the screening procedures.

### Patients

Initially, entry criteria for this study were similar to those of previous efalizumab Phase III trials [14-18]. All patients enrolled were 18 to 75 years of age with at least a 6-month history of plaque psoriasis, with involvement of ≥10% of total body surface area (BSA), had a minimum PASI of 12.0 at screening, and had received previous systemic treatment for psoriasis or were treatment-naïve candidates for such therapy. Patients experiencing clinically significant disease flare at screening or enrollment were excluded, as were patients with a major concomitant illness, immune disorder, or organ dysfunction. During the study, a protocol amendment was introduced that restricted enrollment to patients meeting the High-Need criteria (defined above).

### Patient-reported outcome assessments

The effect of efalizumab on quality of life in patients with moderate to severe plaque psoriasis was assessed using 2 HRQOL instruments: a general health questionnaire (the Short Form-36 [SF-36]) and a validated, self-administered, dermatology-specific questionnaire (the DLQI). Patients evaluated psoriasis symptoms using the Psoriasis Symptom Assessment (PSA) and an itching visual analog scale (VAS). In addition, patients evaluated the overall severity of their psoriasis using the Patient's Global Psoriasis Assessment (PGPA). Patient-reported outcome assessments were performed at baseline and Weeks 4, 8, and 12.

The SF-36 is a multipurpose health survey that measures functional health and well-being in 8 dimensions: Mental Health (psychological distress and well-being), Pain (bodily pain), Physical Functioning (limitations in physical activities because of health problems), Role Emotional (limitations in usual activities because of emotional problems), Role Physical (limitations in usual activities because of physical problems), Social (limitations in social activities because of physical or emotional problems), Vitality (energy or fatigue), and General Health Perceptions [21]. Scores for each dimension range from 0 to 100, with higher scores representing better quality of life. The 8 subscales can be combined to produce 2 summary measures, called the Physical Health and Mental Health scores. The Physical Health score is the sum of the subscale scores for Physical Function, Role Physical, Pain, and General Health Perceptions; and the Mental Health score is the sum of the subscale scores for Vitality, Social, Role Emotional, and General Mental Health. The Physical Health and Mental Health scores can therefore range from 0 to 400, with higher scores representing better health perceptions. An overall summary score is also produced, which consists of the sum of all the subscale scores (possible scores range from 0 to 800).

The DLQI is a reliable, validated 10-item questionnaire covering 6 dimensions (symptoms and feelings, daily activities, leisure, work and school, personal relationships, and treatment) that assesses the overall impact of skin disorders and current treatments on the patient's functioning and well-being [22]. Each question has 4 possible responses, with lower scores representing a better quality of life.

The PSA is a psoriasis-specific questionnaire derived from the symptom scale of the Skindex-29, a validated skin disorder instrument [23], consisting of a 16-item patient-reported measure of 8 psoriasis-related cutaneous symptoms: pain, burning or stinging, itching, bothered by water, irritation, sensitivity, bleeding, and scaling. The frequency and severity of each symptom over the preceding 2 weeks are reported on a scale from 0 to 3, corresponding to categories ranging from "never" to "always," respectively, for frequency, and "not at all" to "a great deal" for severity. Individual scores are summed to give the values of the 2 PSA component subscales that evaluate overall frequency and severity of disease symptoms, with lower scores representing less frequent or less severe psoriasis symptoms.

The itching scale used in this study measures the severity of itching at a specific point in time, using a horizontal VAS. Possible scores range from 0 (no itching) to 10 (severe itching).

The PGPA consists of a single self-explanatory item to be completed by the patient, evaluating overall cutaneous disease at a specific point in time, with possible scores ranging from 0 (no psoriasis) to 10 (worst psoriasis imaginable).

### Statistical analyses

This study was designed to evaluate the efficacy and safety of efalizumab compared to placebo in patients with moderate to severe plaque psoriasis. A protocol amendment introduced assessment of efficacy and safety in the High-Need cohort as well as the total study population, changing entry criteria to ensure enrollment of sufficient High-Need patients. The same amendment added an interim analysis to determine efficacy in the originally defined study population and to verify that the study had adequate power to detect relevant treatment differences in the High-Need population, using the primary endpoint of PASI-75 (Dubertret L, Sterry W, Bos JD, *et al.*, unpublished data).

The intent-to-treat population, consisting of all randomized patients who received at least 1 dose, was the primary population for all efficacy analyses, including patient-reported outcomes. For patients missing a Week 12 value, the last observed value was carried forward to impute missing data (last observation carried forward imputation). Statistical analyses on continuous parameters were performed using analysis of variance (ANOVA) on raw data, adjusting for baseline PASI (≤16.0, ≥16.1), previous use of systemic treatment for psoriasis (yes or no), and geographical region. Analyses were performed on transformed data (e.g., log transformed or ranked) if assumptions of normality were not satisfied. Patients were included in analyses of patient-reported outcomes only if questionnaires were completed with sufficient information to calculate the scores and improvements from baseline. The SF-36 and DLQI were not evaluated in patients from centers where appropriate validated translations of the questionnaire were unavailable (the Czech Republic, Greece, Portugal, and Russia for the SF-36; Greece, Israel, Portugal, and Russia for the DLQI).

## Results

### Patient disposition

A total of 793 patients with moderate to severe plaque psoriasis were randomized and received at least 1 dose of efalizumab (n = 529) or placebo (n = 264). The study population included a High-Need cohort of 526 patients, of whom 342 were randomized to efalizumab and 184 to placebo. Demographic and baseline disease characteristics were comparable between treatment groups in both the High-Need cohort and the total study population, as previously described (Dubertret L, Sterry W, Bos JD, *et al.*, unpublished data).

### Patient-reported measures of HRQOL

#### Short Form-36

**Figure 1:**
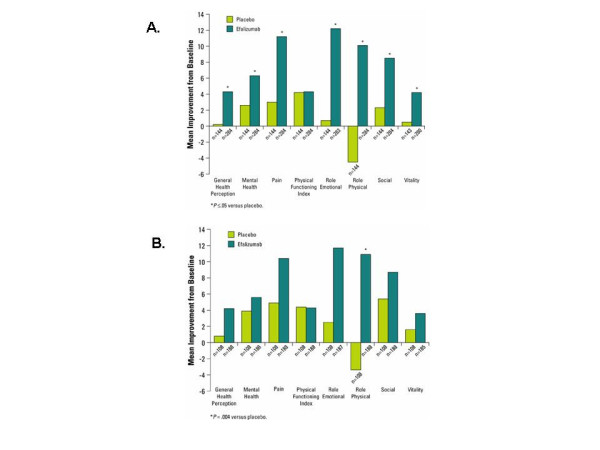
Mean improvement from baseline in Short Form-36 components at Week 12 in the total (A) and High-Need (B) study populations.

At Week 12, efalizumab-treated patients showed significantly greater improvements from baseline than did placebo patients (*P *≤ .05) in each SF-36 component except for the Physical Functioning Index (Figure 1A). It should be noted that the baseline scores for the Physical Functioning Index were highest among all the SF-36 dimensions for all patients (mean ± SD = 77.8 ± 25.3, as compared to a mean ± SD range from low to high of 55.3 ± 20.5 for Vitality Index to 64.7 ± 40.9 for Role Physical). The high baseline score for the Physical Functioning Index indicates that impairment of quality of life was not as large on this dimension as compared to the other dimensions. The greatest differences in improvement associated with efalizumab therapy compared to placebo were in the domains that measure role limitations due to physical and emotional health, the Role Physical (10.1 points for efalizumab vs. -4.5 points for placebo; *P *< .001) and Role Emotional (12.2 points efalizumab vs. 0.7 points placebo, *P *= .005) dimensions. The SF-36 overall summary score improved by 59.7 points for patients receiving efalizumab versus 10.4 points for patients receiving placebo (*P *= .002). As in the total population analysis, efalizumab-treated High-Need patients demonstrated greater mean improvements from baseline than placebo patients in all SF-36 dimensions, except for the Physical Functioning Index, with the greatest difference in improvement in the Role Physical (10.9 vs -3.4, respectively, *P *= .004) and Role Emotional domains (11.7 points vs 2.5 points, respectively, *P *= .004; Figure 1B). Overall, efalizumab-treated High-Need patients demonstrated significantly greater improvement from baseline compared to placebo in the SF-36 summary score (57.3 points efalizumab vs. 19.7 points placebo, *P *= .039).

#### Dermatology Life Quality Index

**Figure 2:**
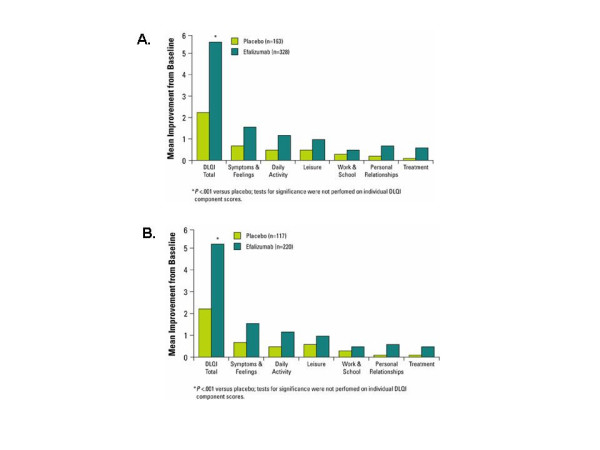
Mean improvement from baseline in Dermatology Life Quality Index components at Week 12 in the total (A) and High-Need (B) study populations.

At Week 12, patients in the efalizumab group demonstrated a significantly greater improvement from baseline in DLQI total score compared with placebo patients (5.7 points vs. 2.3 points, respectively; *P *< .001) (Figure 2A). Patients receiving efalizumab had greater improvements from baseline relative to placebo in all 6 dimensions of the DLQI, with the largest improvement observed in the Symptoms and Feelings dimension (1.6 points for efalizumab vs. 0.7 points for placebo). Improvement from baseline in DLQI total score was significantly greater for efalizumab than placebo as early as Week 4 (*P *< .001). Results at Week 12 were similar between the High-Need cohort and the total study population, with efalizumab-treated patients achieving significantly greater improvement than placebo-treated patients in DLQI total score (5.4 points vs 2.3 points, respectively, *P *< .001; Figure 2B). Greater improvements from baseline were evident for efalizumab-treated patients compared to placebo-treated patients for all individual components of the DLQI. As observed in the total study population, in the High-Need cohort, improvement from baseline in DLQI total score was significantly greater with efalizumab compared to placebo as early as Week 4 (*P *< .001). Further, the largest improvement in the High-Need patients was observed in the Symptoms and Feelings dimension and was identical to that observed in the total study population (Figure 2B).

#### Psoriasis Symptom Assessment

At Week 12, improvements in the PSA Frequency and Severity scores in the total study population were significantly greater for patients in the efalizumab group compared to the placebo group. PSA Frequency had improved by 5.7 points in efalizumab-treated patients versus 2.0 points for placebo-treated patients, and PSA Severity had improved by 6.2 points in the efalizumab group versus 1.9 in the placebo group (*P *< .001 for both analyses; Table 1). A significant difference between treatments was observed as early as Week 4 (*P *< .001). Results for the High-Need cohort were comparable to those observed in the total study population (Table 1). High-Need efalizumab-treated patients achieved significantly greater improvement than placebo-treated patients in the PSA Frequency (5.8 points vs 2.1 points, *P *< .001) and PSA Severity (6.3 points vs 1.9 points, *P *< .001) scores (Table 1).

**Table 1:** Mean improvement from baseline at Week 12 for patient-reported clinical outcome measures in the total study population and the High-Need cohort.

	**PSA**			**Itching VAS**		**PGPA**	
**Patient Population**	n	**Frequency **Mean	**Severity **Mean	n	Mean	n	Mean

**High-Need Cohort**							
Placebo	184	2.1	1.9	184	0.4	182	0.4
Efalizumab	340	5.8*	6.3*	339	2.4*	341	2.8*
**Total Population**							
Placebo	262	2.0	1.9	261	0.6	261	0.4
Efalizumab	527	5.7*	6.2*	526	2.5*	528	2.8*

*P < .001 versus placebo.

PSA, Psoriasis Symptom Assessment; VAS, Visual Analog Scale; PGPA, Patient's Global Psoriasis Assessment.

#### Itching Visual Analog Scale

In the total study population, the mean itching VAS score had improved from baseline by 2.5 points from baseline at Week 12 in efalizumab-treated patients, compared to 0.6 points for placebo-treated patients (*P *< .001; Table 1). A significant difference between treatment groups in mean improvement from baseline was observed as early as Week 4 (*P *< .001). As in the total study population, efalizumab patients in the High-Need cohort demonstrated significantly greater improvement than placebo patients from baseline at Week 12 (2.4 points vs 0.4 points, *P *< .001; Table 1) with significant improvement noted as early as Week 4 (*P *< .001).

#### Patient's Global Psoriasis Assessment

In the total study population, greater mean improvements from baseline were observed in efalizumab-treated patients compared to placebo-treated patients at Week 12 (2.8 points efalizumab vs. 0.4 points placebo, *P *< .001; Table 1). Identical improvements were observed in the High-Need cohort (Table 1).

### Safety and tolerability

The safety profile of the 12-week course of efalizumab in this trial is reported elsewhere (Dubertret L, Sterry W, Bos JD, *et al.*, unpublished data). Briefly, adverse events were generally mild to moderate in severity and were similar between the total study population and the High-Need cohort. In the total study population, the most frequently reported adverse events (≥5% of patients in either treatment group) were headache (reported in 26.1% of efalizumab vs. 14.0% of placebo patients), "influenza-like illness" (9.6% vs. 7.2%), arthralgia (7.4% vs. 3.0%), rigors (6.2% vs. 5.3%), pyrexia (7.9% vs. 1.1%), nasopharyngitis (5.3% vs. 4.2%), myalgia (5.5% vs. 2.7%), and pruritus (3.6% vs. 5.7%). Serious adverse events were reported in 5.5% of efalizumab-treated patients and 3.4% of placebo patients in the total study population. Adverse events led to withdrawal in 5.7% of efalizumab and 2.7% of placebo patients.

## Discussion

This randomized, placebo-controlled trial of efalizumab in patients with moderate to severe plaque psoriasis is the first to evaluate the impact of a psoriasis therapy on patient-reported outcomes in a large cohort of prospectively, but subjectively, defined High-Need patients. Enrollment of High-Need patients allowed assessment of a new treatment option for a patient group that can be particularly challenging to clinicians.

In this study, 12 weeks of efalizumab treatment resulted in significant improvements in multiple patient-reported outcomes including 2 HRQOL instruments (SF-36 and DLQI) and patient-reported efficacy measures (PSA, itching VAS, and PGPA), in both the total study population and the High-Need cohort. These results confirm and extend findings reported from several Phase III efalizumab trials [20]. Importantly, results for the CLEAR trial's High-Need cohort, a difficult-to-treat population, demonstrated similar improvements to those seen in the broader group of moderate to severe psoriasis patients and in previous efalizumab studies [20]. Improvements from baseline in PSA Frequency, PSA Severity, itching VAS, and PGPA were statistically significantly greater for efalizumab-treated High-Need patients than for placebo-treated High-Need patients. Collectively, these results suggest that, as assessed by the patients themselves, efalizumab treatment results in improvements in multiple aspects of psoriasis symptoms, including the severity and frequency of pruritus, pain, bleeding, scaling, and irritation, and in the impact of these symptoms on patients' daily lives and mental health.

Efalizumab-treated High-Need patients achieved significantly greater overall quality-of-life improvement than placebo-treated patients as measured by all individual components of the dermatology-specific DLQI and in most SF-36 dimensions. With the exception of the Role Physical dimension (measuring role limitations due to physical health), treatment differences in the individual dimensions were not statistically significant for the High-Need population. The reduction in evaluable patients for the SF-36 (and DLQI) due to absence of validated questionnaires in some countries might have contributed to the absence of statistically significant differences in SF-36 components between treatment groups in the High-Need cohort. The significant effect of efalizumab treatment on physical functioning indicates the negative impact psoriasis has on a patient's physical abilities as they relate to the frequency at which physical activities are performed; thus Role Physical is well suited for evaluating an aspect of patient QOL that is impacted by psoriasis.

Recent critical review of the DLQI and SF-36 quality-of-life measures for validity and internal consistency has demonstrated high reproducibility and internal consistency scores for both scales [24]; however, the relationship to a clinical meaningful response requires further analysis. A previous analysis of patient-reported outcome measures including the DLQI, PSA, and 2 itch scales from 2 randomized clinical trials of efalizumab demonstrated significant correlation with 2 measures of clinical outcome, PASI and Overall Lesion Severity (OLS) scores [25]. More recently, the clinical relevance of improvement in QOL was evaluated in a pooled analysis of 4 Phase III, randomized, double-blind, placebo-controlled studies of efalizumab (Finlay, et al., unpublished results presented at the 3rd EADV Spring Symposium, Sofia, Bulgaria, May 19–22, 2005). The analysis employed a minimal clinically important difference (MCID) that was defined as a change in DLQI of ≥5 points [26]; 57% of efalizumab-treated patients achieved MCID after 12 weeks of therapy compared with 29% of placebo-treated patients. Under these criteria for MCID, the mean improvement in DLQI for the total study population and High-Need cohort indicates achievement of MCID in the present study.

A mean Physical Functioning Index of 84.2 was found from a general US population [27], supporting the conclusion that the baseline score for our study (77.8) was already near the maximum score typically observed. The relatively high patient scores for the Physical Functioning Index at baseline compared to the other SF-36 dimensions suggest that this dimension may not be particularly sensitive to the aspects of HRQOL affected by psoriasis. These findings demonstrate the importance of assessing quality of life in psoriasis using a combination of a general instrument and a dermatology-specific instrument [28]. Together, the results from the SF-36 and the DLQI demonstrate that efalizumab treatment results in improvements in both physical and mental aspects of HRQOL in both the total study population and the High-Need cohort.

Systemic psoriasis therapies, such as methotrexate, cyclosporine, acitretin, and psoralen-ultraviolet A (PUVA) phototherapy, are efficacious in improving PASI and HRQOL with short-term use [10,29]. However, these agents are associated with toxicities or contraindications that may limit their long-term use and make them unsuitable for many patients [30-33]. With efalizumab, rapid and significant improvements in HRQOL and in physician-assessed (Dubertret L, Sterry W, Bos JD, *et al.*, unpublished data) and patient-assessed efficacy measures were observed in the High-Need cohort of this study, for whom several other systemic agents were not viable treatment options. The safety profile was similar between the High-Need cohort and the total study population and was consistent with that observed in previous Phase III randomized efalizumab studies [14-18]. Furthermore, results from an open-label Phase III study of up to 36 months of continuous efalizumab therapy in psoriasis suggest that the efficacy and safety profiles of efalizumab are maintained during extended treatment (Leonardi CL, *et al.*, unpublished data). Three-year safety and efficacy data are particularly important considering that current guidelines recommend against continuous use of cyclosporine and recommend periodic liver biopsies with continued methotrexate use [31,33]. The significant improvements achieved by High-Need patients in the present study, combined with the favorable safety profile demonstrated in this cohort, suggest that efalizumab therapy may fulfill the need for a viable therapeutic option in this patient population.

## Conclusion

In summary, these results demonstrate that efalizumab provided significant patient-reported improvements in HRQOL, in terms of both the frequency and severity of psoriasis symptoms and their impact on the patient's daily life and sense of well-being. These improvements were observed in both the total study population of patients with moderate to severe plaque psoriasis and the High-Need cohort for whom other systemic therapies were unsuitable. The HRQOL findings support and reinforce the findings of efficacy according to physician-assessed measures such as the PASI (Dubertret L, Sterry W, Bos JD, *et al.*, unpublished data). Moreover, a favorable safety profile was demonstrated in both study populations. Given the negative impact that psoriasis can have on HRQOL, these are important findings. The results indicate that the beneficial impact of efalizumab on QOL is consistent regardless of disease severity, prior therapy, or contraindications to previous therapies and are particularly encouraging as they suggest the possibility of effective treatment in High-Need, or difficult-to-treat, patients who have few viable treatment options.

## Competing interests

JPO is a consultant and member of the speakers bureau for, and has received research support from, Serono International S.A. NS is a consultant and member of the speakers bureau for, and has received research support from, Serono International SA. SS has received research support from Serono International SA. EH is an employee and stockholder of Serono International SA.

## Authors' contributions

All authors contributed equally to the development of this manuscript. JPO, NS, and SS enrolled and examined patients during the course of the study. EH planned and carried out the data analysis. All authors read and approved the final manuscript.

## Pre-publication history

The pre-publication history for this paper can be accessed here:



## Supplementary Material

Additional File 1 Word file listing the CLEAR (Clinical Experience Acquired with Raptiva) trial investigators Click here for file
