# Supplementary material for: Impact of efalizumab on patient-reported outcomes in high-need psoriasis patients: results of the international, randomized, placebo-controlled Phase III Clinical Experience Acquired with Raptiva (CLEAR) trial [NCT00256139]
Source: BMC Dermatol. 2005 Dec 16;5:13. doi: 10.1186/1471-5945-5-13 (PMC1343580; doi:10.1186/1471-5945-5-13)
Supplement: Additional File 1 — Word file listing the CLEAR (Clinical Experience Acquired with Raptiva) trial investigators [file 1471-5945-5-13-S1.doc]

**CLEAR (Clinical Experience Acquired with Raptiva) Trial Investigators**

Dr. G.V. Akovbyan

Dispensary for Skin and Venereal Diseases

Moscow Region Korolyov, Russia

Prof. Dr. Sibel Alper

Ege University

Bornova-Izmir, Turkey

Prof. Christina Antoniou

University of Athens

Athens, Greece

Prof. Petr Arenberger

Dermatology Clinic

Praha, Czech Republic

Prof. Dr. Nilgun Atakan

Hacettepe University

Sihhiye Ankara, Turkey

Prof. Dr. Gulsevim Azizlerli

Istanbul University

Capa Istanbul, Turkey

Prof. Christopher Bedane

CHU Hôpital Dupuytren

Limoges, France

Prof. Jean-Jacques Bonerandi

CHU Hôpital La Timone

Marseille, France

Prof. Jan D. Bos

University of Amsterdam

Amsterdam, Netherlands

Dr. Marc Bourcier

Dermatology Clinic

Moncton, New Brunswick, Canada

Prof. Dr. Gunter Burg

Universitats Spital Zurich

Zurich, Switzerland

Dr. Jorge Cardoso

Hospital Curry Cabral

Lisboa, Portugal

Dr. Mariano Casado Jiménez

Hospital Universitario La Paz

Madrid, Spain

Prof. Sergio Chimenti

Policlinico Universitario Tor Vergata

Roma, Italy

Prof. Alain Claudy

Hôpital Edouard Herriot

Lyon, France

Dr. Peter Cowen

Private Consulting

Clayton, Victoria, Australia

Dr. Morten Dalaker

St. Olavs Hospital

Trondheim, Norway

Dr. Esteban Dauden

Hospital Universitario de La Princesa

Madrid, Spain

Prof. Michael David

Rabin Medical Center

Petach Tikva, Israel

Prof. Michel de la Brassinne

Domaine Universitaire du Sart Tilman

Liege, Belgium

Prof. Louis Dubertret

Hôpital Saint Louis

Paris, France

Prof. Dr. med. Peter Elsner

Friedrich-Schiller University of Jena

Jena, Germany

Dr. Tulin Ergun

Marmara Universitesi Tip Fakültesi

Istanbul Altunizade, Turkey

Dr. Vladimir V. Evstafiev

Smolensk State Medical Academy

Smolensk, Russia

Dr. Carlos Ferrándiz-Foraster

Hospital Universitari Germans Trias i Pujol

Badalona (Barcelona), Spain

Prof. Dr. med. Gerhard Fierlbeck

Universitätsklinikum

Tübingen, Germany

Prof. Américo Figueiredo

Hospitais da Universidade de Coimbra

Coimbra, Portugal

Prof. Dr. Peter Fritsch

Vorstand der Univ.-Klinik für Dermatologie u. Venerologie

Innsbruck, Austria

Prof. Alberto Giannetti

Struttura Complessa di Dermatologia

Modena, Italy

Dr. José Fernando Barba Gómez

Instituto Dermatológico de Jalisco “Dr. José Barba Rubio”

Jalisco, Mexico

Dr. Minerva Gómez Flores

Hospital Universitario “Dr. José Eleuterio González”

Monterrey, N.L., Mexico

Dr. Remigio González Soto

Centro de Dermatología de Monterrey

Monterrey, N.L., Mexico

Dr. David Gratton

International Dermatology Research, Inc.

Montreal, Quebec, Canada

Dr. Mario Amaya Guerra

Hospital de Especialidades

Monterrey, N.L., Mexico

Dr. Jean-Jacques Guilhou

CHU Hôpital St Eloi

Montpellier, France

Prof. Gérard Guillet

CHU Hôpital Jean Bernard Miletrie

Poitiers, France

Prof. Michel Heenen

Hôpital Erasme

Brussels, Belgium

Dr. Philippe Humbert

CHU Hôpital St Jacques

Besançon, France

Dr. Nilsel Ilter

Gazi University

Ankara Besevler, Turkey

Prof. Arieh Ingber

Hadassah Ein Karem

Jerusalem, Israel

Prim. Univ. Prof. Dr. Wolfgang Jurecka

Wilhelminenspital

Wien, Austria

Prof. Dr. Helmut Kerl

Vorstand der Univ.-Klinik für Dermatologie u. Venerologie

Graz, Austria

Dr. I.A. Klemenova

Nizhegorodskiy Scientific Institute for Skin and Venereal Diseases

N. Novgorod, Russia

Prof. Nikolay G. Korotkiy

Russian State Medical University

Moscow, Russia

Prof. Anna A. Koubanova

Central Research Institute for Skin and Venereal Diseases

Moscow, Russia

Prof. N.V. Kungurov

Uralski Scientific Institute of Dermatology and Immunology MoH

Ekaterinburg, Russia

Dr. Richard G.B. Langley

Eastern Canada Cutaneous Research Associates Inc.

Halifax, Nova Scotia, Canada

Dr. Christian Gronhoj Larsen

Arhus universitetssjukhus

Aarhus C, Denmark

Dr. M. Lecha Carralero

Servicio de Dermatologia, Hospital Clinic

Barcelona, Spain

Dr. Gladys León Dorantes

Hospital General de Mexico

Mexico DF, Mexico

Prof. Dr. Thomas A. Luger

Dermatologische Klinik der Westfaelischen

Münster, Germany

Dr. Charles Lynde

Lynde Center for Dermatology

Markham, Ontario, Canada

Dr. Cem Mat

Istanbul University

Istanbul Cerrahpasa, Turkey

Dr. Christopher McCormack

Emeritus Research

Malverne, Victoria, Australia

Dr. Benjamin Moncada González

Hospital Central “Dr. Ignacio Morones Prieto”

San Lui Potosi, Mexico

Dr. Dagfinn Moseng

University Hospital of North Norway

Tromso, Norway

Dr. Olga Mourellou-Tsatsou

Hospital of Dermatological & Venereal Diseases of Thessaloniki

Thessaloniki, Greece

Prof. Dr. Ulrich Mrowietz

University of Schleswig-Holstein

Kiel, Germany

Prof. Luigi Naldi

Ospedali Riuniti di Bergamo

Bergamo, Italy

Dr. N.M. Olekhnovich

Saratov State Medical University

Saratov, Russia

Asst. Prof. Dr. Nahide Onsun

SSK Vakif Gureba Hospital

Fatih Istanbul, Turkey

Prof. Jean-Paul Ortonne

CHU Hôpital l’Archet

Nice, France

Dr. Kim Papp

Probity Medical Research

Waterloo, Ontario, Canada

Prof. SSA Ketty Peris

Universita’ degli Studi di L’Aquila

L’Aquila, Italy

Prof. Yuri N. Perlamutrov

City Dispensary for Skin and Venereal Diseases #7

Moscow, Russia

Dr. Jordi Peyri Rey

Hospital Principes de España (H. Bellvitge)

Barcelona, Spain

Prof. António Pinto Soares

Hospital do Desterro

Lisboa, Portugal

Prof. Dr. med. Dr. h.c. Gerd Plewig

University of Munich

Munich, Germany

Prof. Nikolay N. Potekaev

City Dispensary for Skin and Venereal Diseases #4

Moscow, Russia

Dr. Yves P. Poulin

Centre Dermatologique du Quebec Metro

Sainte-Fay, Quebec, Canada

Dr. A.L. Pozdnyakov

Region Dispensary for Skin and Venereal Diseases

Voronezh, Russia

Dr. Ramon Pujol

Hospital del Mar

Barcelona, Spain

Prof. Prim. Dr. Klemens Rappersberger

Vorstand der Dermatologischen Abteilung

Wien, Austria

Prof. K.I. Raznatovsky

St. Petersburg Medical Academy of Postgraduate Education

St. Petersburg, Russia

Prof. Carlos Resende

Hospital de S. João

Porto, Portugal

Prof. Jean Revuz

CH Henri Mondor

Créteil, France

Prof. Dr. med. Dr. phil. Johannes Ring

Technische Universität München

Munich, Germany

Prof. Jorgen R. Ronnevig

Rikshospitalet

Oslo, Norway

Prof. A.V. Samtsov

Military Medical Academy

St. Petersburg, Russia

Prof. Jean-Hilaire Saurat

Hôpitaux Universitaires de Genève

Geneva, Switzerland

Dr. Jean-Luc Schmutz

CHU Hôpital Maringer-Villemin-Fournier

Nancy, France

Asst. Prof. Dr. Deniz Seçkin

Baskent University Medical Faculty Hospital

Ankara, Turkey

Dr. Neil H. Shear

Ventana Clinical Research

Toronto, Ontario, Canada

Dr. Stephen Shumack

St George Dermatology

Kogarah, Australia

Dr. Rodney Sinclair

The Alfred Hospital

Prahran, Australia

Prof. Pierre Souteyrand

CHU Hôtel Dieu

Clermont Ferrand, France

Prof. Dr. Wolfram Sterry

University Hospital Charité

Berlin, Germany

Prof. Dr. Georg Stingl

Abteilung für Immundermatologie und Infektiöse Hautkrankheiten

Wien, Austria

Dr. L.P. Sukhova

City Dispensary for Skin and Venereal Diseases

Lipetsk, Russia

Dr. Phillip Swarbrick

Burswood Dermatology

Victoria Park, Australia

Dr. Alain Taieb

Hôpital Saint-André

Bordeaux, France

Dr. Jerry Kim Tan

Windsor Clinical Research Inc.

Windsor, Ontario

Prof. Pierre Thomas

Hôpital Cardiologique

Lille, France

Dr. A.L. Tischenko

Russian People Friendship University

Moscow, Russia

Dr. Zohair Tomi

New Lab Clinical Research Inc.

St. John’s, Newfoundland, Canada

Prof. Jaime Toribio

Hospital Gil Casares

Santiago de Compostela, Spain

Prof. Henry Trau

Sheba Hospital

Tel Hashomer, Israel

Prof. Dr. Uksal Ümit

Erciyes University Medical Faculty Hospital

Kayseri, Turkey

Asst. Prof. Vladimir Vasku

Faculty Hospital U Sv. Anny

Brno, Czech Republic

Dr. Ronald B. Vender

Dermatology Centre

Hamilton, Ontario, Canada

Dr. Glenda Wood

Prince of Wales Hospital

Randwick, Australia

Prof. V.V. Yakusevich

Soloviev’s Clinical Hospital for Urgent Medical Care

Yaroslavl, Russia

Dr. Hugh Zacharie

Hojbjerg, Denmark
